# Supplementary material for: Synthetic photosynthetic consortia define interactions leading to robustness and photoproduction
Source: J Biol Eng. 2017 Jan 23;11:4. doi: 10.1186/s13036-017-0048-5 (PMC5259876; doi:10.1186/s13036-017-0048-5)
Supplement: Additional file 1: — Contains supplementary figures S1-S15 as well as the mathematical framework used to investigate microbial community interactions. (DOCX 75744 kb) [file 13036_2017_48_MOESM1_ESM.docx]

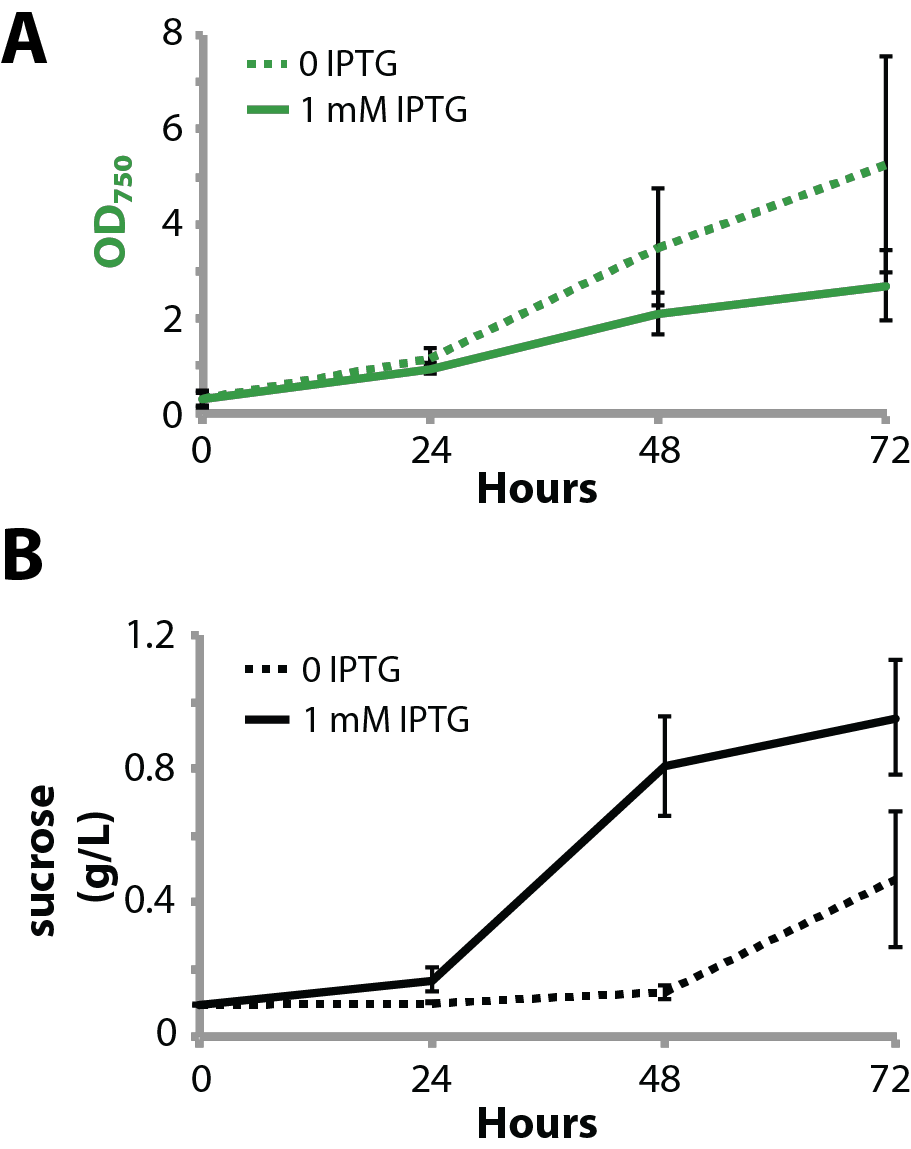


**Fig. S1 Axenic cyanobacteria characterization in ^CoY^BG-11.**

Cell growth of *cscB^+^* *S. elongatus* (**A**) and sucrose levels in culture supernatants (**B**) were measured in ^CoY^BG-11 with (solid lines) or without (dashed lines) IPTG. Error bars are standard deviation of 5 biological replicates.


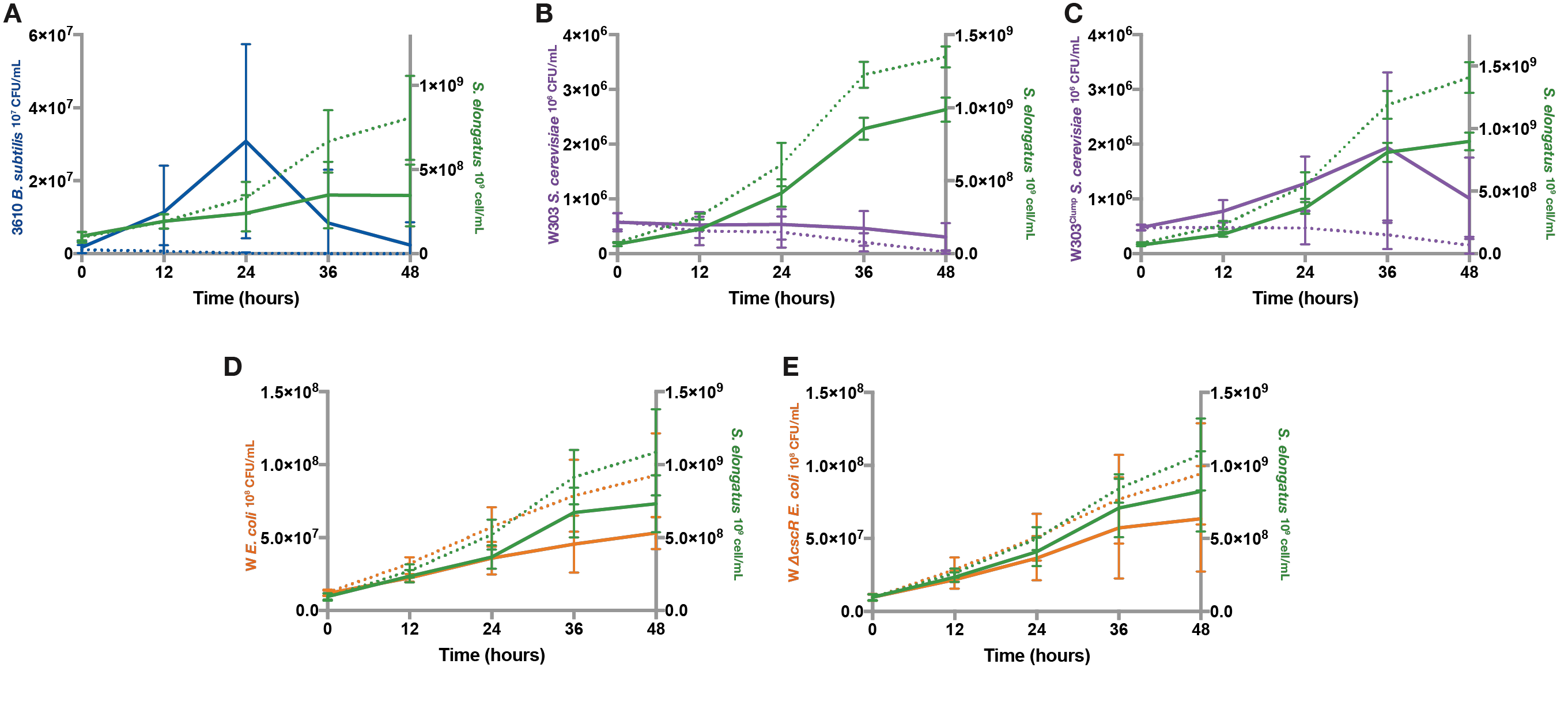


**Fig. S2 Replicate batch co-cultures.**

To capture variation in co-culture growth, 10 biological replicates of the prokaryotic co-cultures (**A, D&E**) and 9 replicates of the *S. cerevisiae* co-cultures (**B, C**) were set up on 5 and 3 different days, respectively. These replicates include those presented in Fig. 2. Error bars represent standard deviation between samples. Cultures with (solid lines) and without (dashed lines) IPTG to induce sucrose secretion are shown. *S. elongatus* (**A-E,** green) was measured in cells/mL in each culture. *B. subtilis* (**A**, blue), *S. cerevisiae* (**B, C,** purple), and *E. coli* (**D**, **E,** orange) were measured via CFU/mL. Two strains of *S. cerevisiae*, the wild type strain W303 (**B**) and W303^Clump^ (**C**) engineered for increased growth in low concentrations of sucrose, were tested. Growth of W303 was not observed however growth of W303^Clump^ was achieved with the addition of IPTG (**C**, solid line). Two strains of *E. coli*, wild type strain W (**D**) and the W Δ*cscR* strain engineered for efficient sucrose utilization (**E**), were tested and consistently showed monotonic growth.


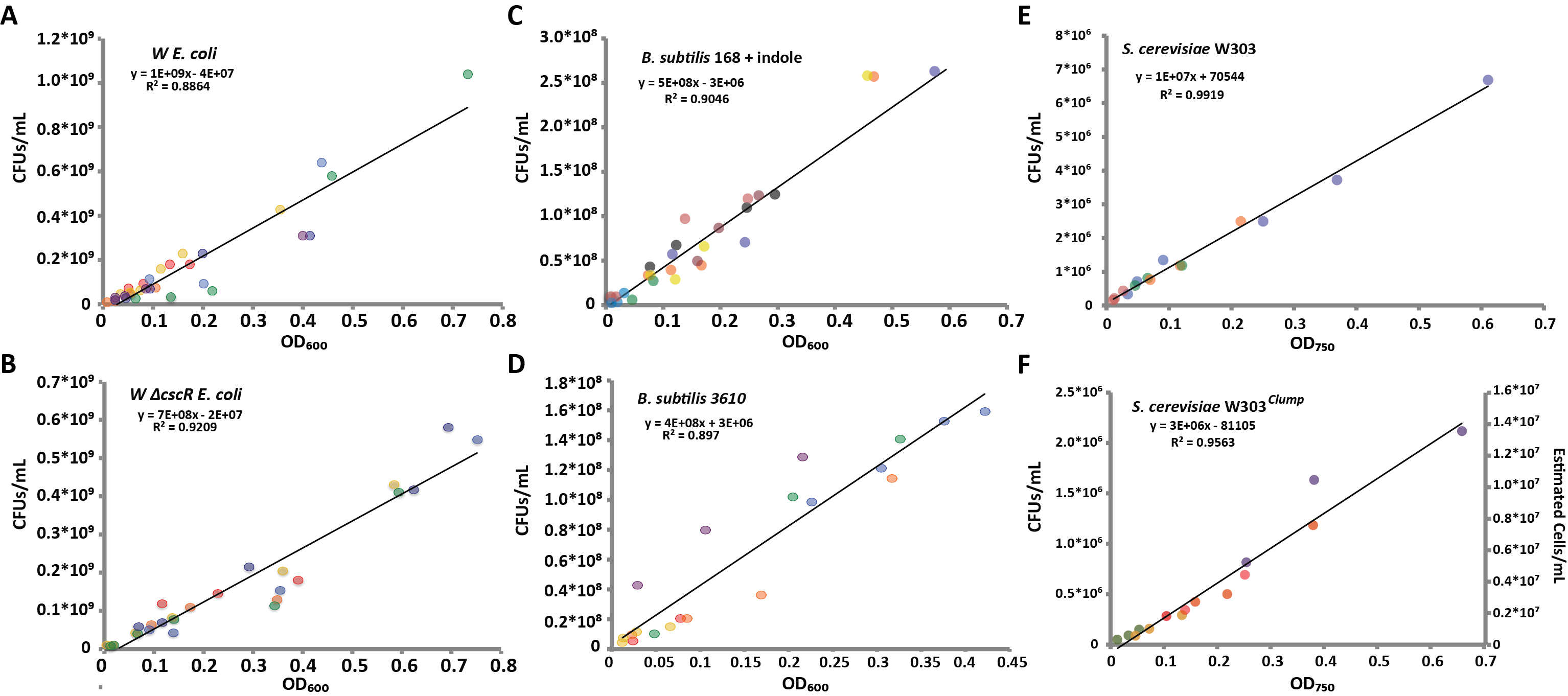


**Fig. S3 Direct relationship between OD_600_ and viable cells counts for all heterotrophs.**

Correlation between OD_600_ and CFU/mL in co-culture media supplemented with 2% sucrose is shown. All strains of heterotrophs were analyzed; *E. coli* W (**A**), *E. coli* W Δ*cscR* (**B**), *B. subtilis* 168 supplemented with indole (**C**), *B. subtilis* 3610 (**D**), *S. cerevisiae* W303 (**E**), and *S. cerevisiae* W303^Clump^ (**F**). Distinct colors represent different biological replicates.


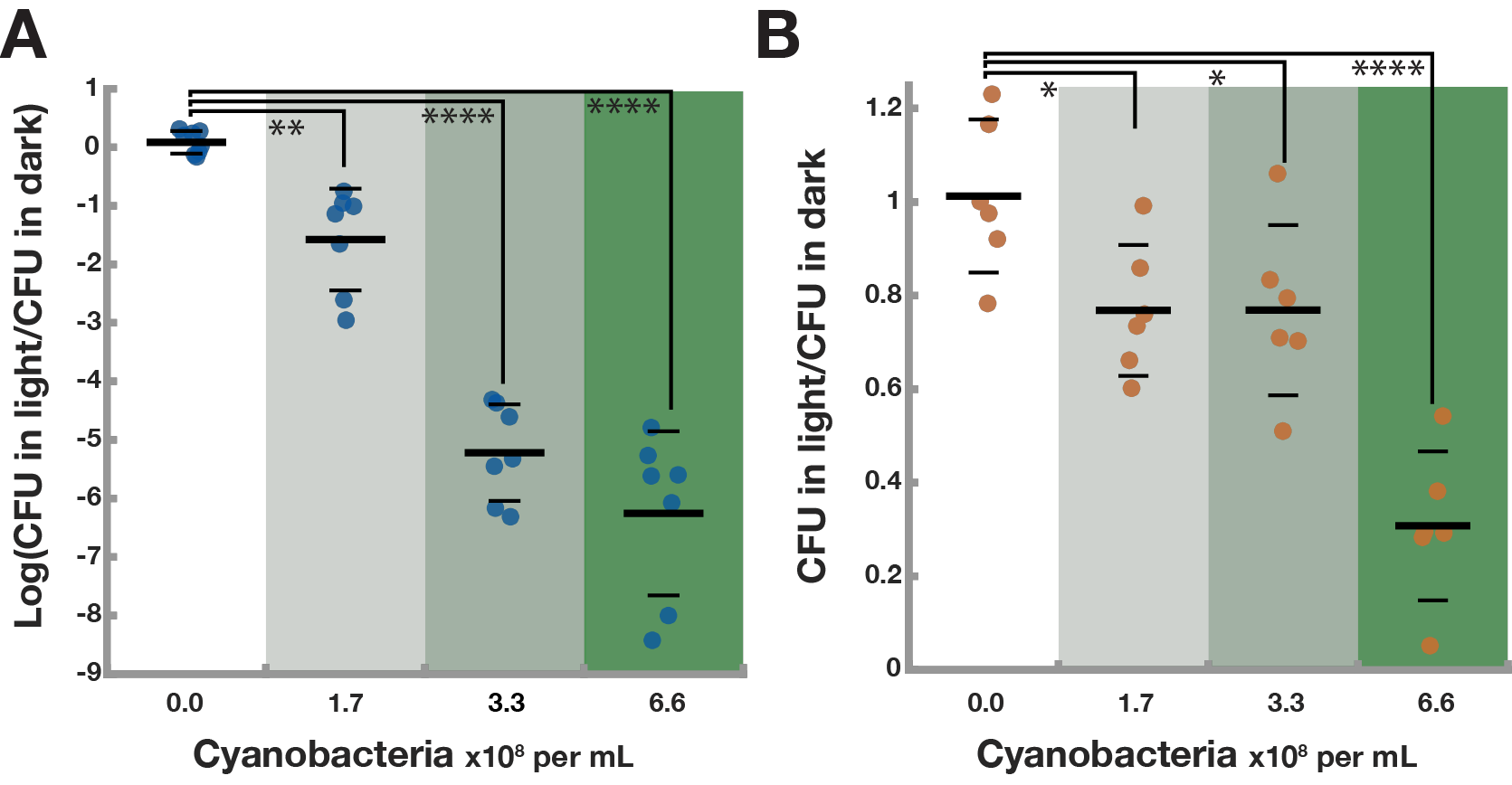


**Fig. S4 Additional heterotrophic prokaryotes demonstrate sensitivity to *S. elongatus* in the light.**

The *B. subtilis* production strain, 168, (**A**) and W *E. coli* strain (**B**) demonstrate light-dependent sensitivity to dense cyanobacteria cultures. Co-cultures were set up with *S. elongatus* of varying concentrations (increasingly dark green backgrounds) and heterotroph CFUs were determined after 12 hours of exposure to either light or dark. Ratios of CFU in light compared to CFU in dark are reported. Thick horizontal lines represent the average measurement for each condition while thin horizontal lines represent one standard deviation from the mean. P-values of two-tailed t-tests with Welch’s correction are denoted with asterisks: * 0.01 to 0.05, ** 0.001 to 0.01, *** 0.0001 to 0.001, **** <0.0001.


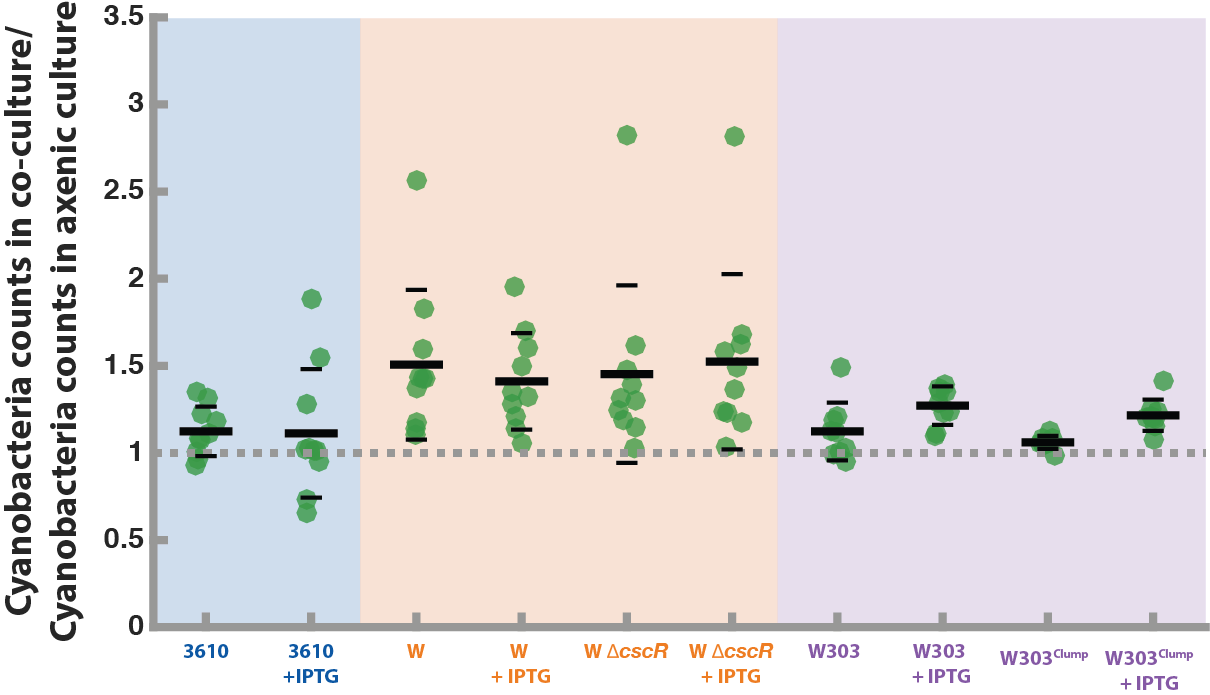


**Fig. S5 Cyanobacteria benefit from heterotrophs in batch culture.**

Results from all batch cultures described in Fig. 2 and Fig. S2 were analyzed by normalizing the number of cyanobacteria in co-cultures to the number of cyanobacteria in axenic control cultures at every data point (every 12 hours for 2 days). Of these points, the maximum value for each culture is plotted (each point is a biological replicate). Averages (thick black lines) and standard deviations (thin black lines) were determined from 10 biological replicates for prokaryotes (*B. subtilis* 3610 in blue, *E. coli*, both WT W and W Δ*cscR,* in orange) and 9 biological replicates for *S. cerevisiae* (purple)*.*


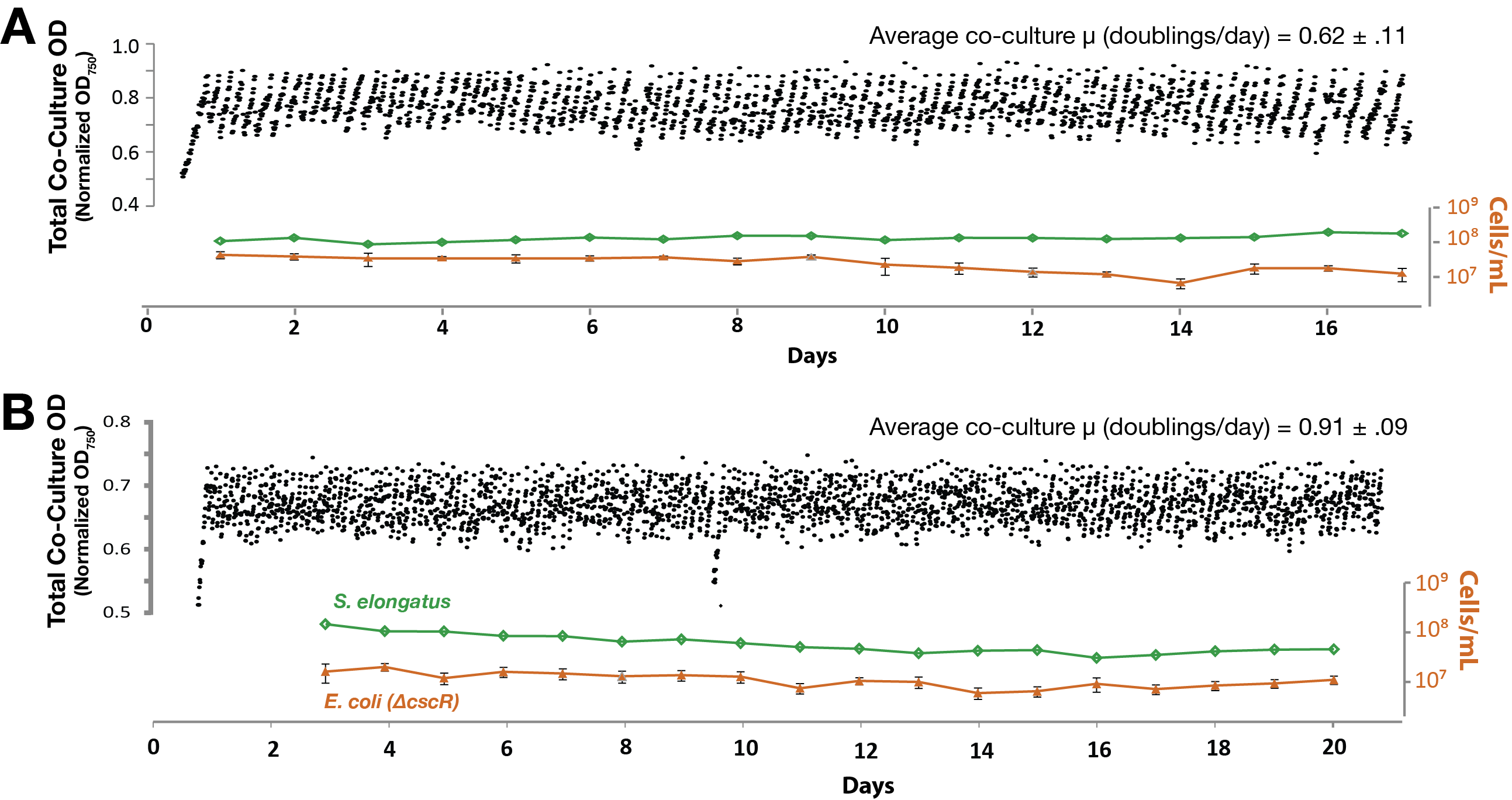


**Fig. S6 Replicate *E. coli/cscB^+^* *S. elongatus* co-cultures.**

Additional photobioreactor continuous co-culture replicates of *E. coli* W Δ*cscR* */cscB^+^* *S. elongatus* with 1 mM IPTG were set up in constant light. Optical density of the entire culture (black scatter plots) and cell counts for the individual species were tracked (green *S. elongatus,* orange *E. coli* W Δ*cscR*) for more than 2 weeks.


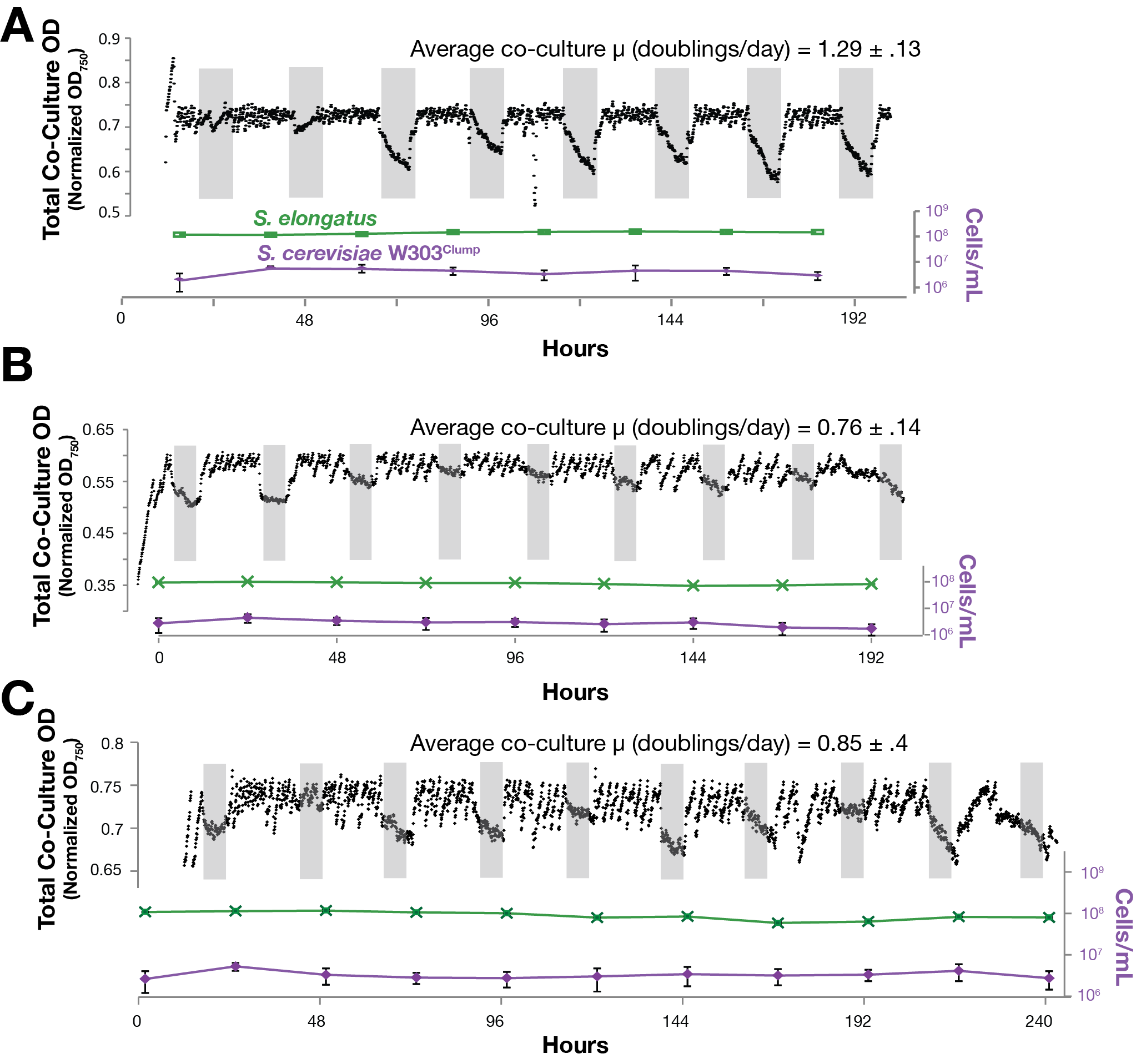


**Fig. S7 Replicate *S. cerevisiae/cscB^+^* *S. elongatus* co-cultures.**

Additional continuous co-culture replicates of W303^Clump^ *S. cerevisiae /cscB^+^* *S. elongatus* with 1 mM IPTG were set up in alternating light/dark (dark periods shown in grey). Optical density of the entire culture (black scatter plots) and cell counts for the individual species were tracked (green *S. elongatus,* purple W303^Clump^ *S. cerevisiae*) for more than a week.


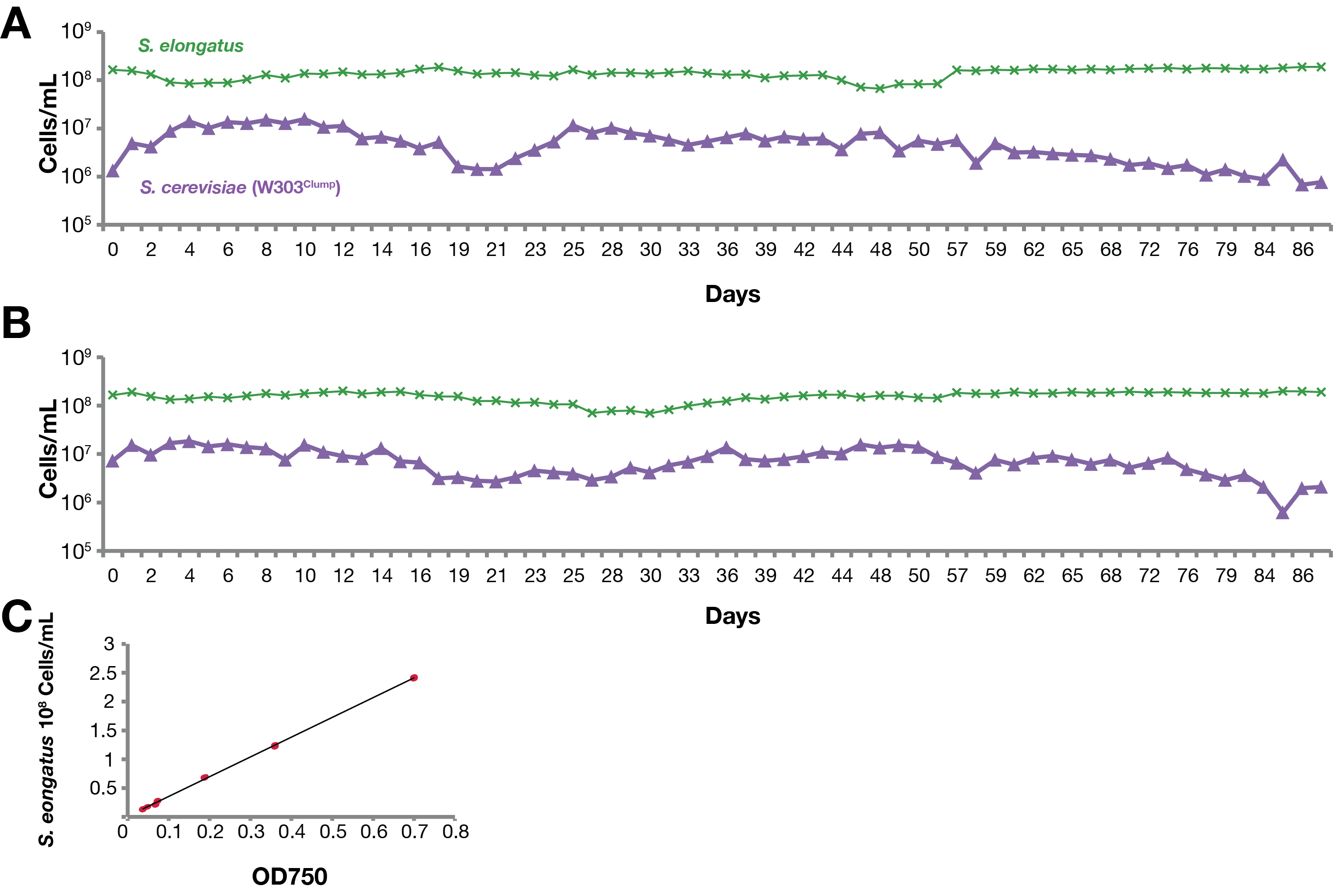


**Fig. S8 Extended continuous *S. cerevisiae/cscB^+^* *S. elongatus* co-cultures.**

Two co-cultures, (**A**) and (**B**), of *S. elongatus* and *S. cerevisiae* W303^Clump^ were inoculated in photobioreactors under constant light with 1mM IPTG to induce sucrose export. The number of cyanobacterial and yeast cells within the co-culture were monitored daily by withdrawing samples from the reactor. W303^Clump^ viable cell counts were determined as for other cultures by plating serial dilutions on YEPD media (light purple). Cell counts for cyanobacteria were estimated by measuring OD_750_ of the total culture following a light centrifugation step (100g for 30 sec) which pelleted >90% of the W303^Clump^ cells but which left smaller cyanobacterial cells in suspension: OD_750_ measurements were compared to a standard curve (**C**) to estimate cyanobacterial cell number (analysis of cyanobacterial cell density in this experiment is distinct from other, later co-culture data collection – where FACS analysis was utilized).


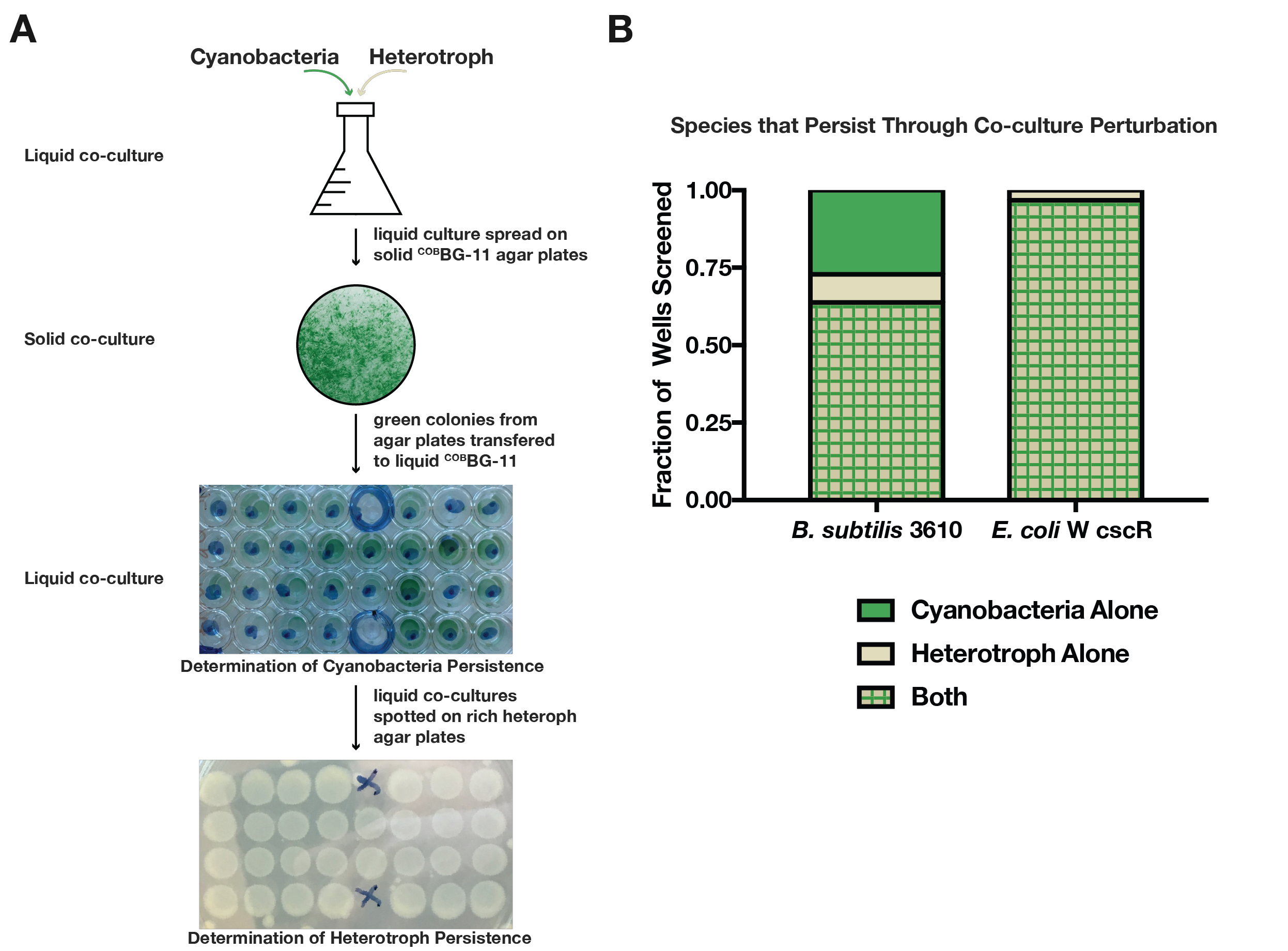


**Fig. S9 Persistence of prokaryotic co-cultures through perturbation of environmental structure.**

**(**A) Liquid co-cultures of *cscB^+^ S. elongatus* with either *B. subtilis* strain 3610 or *E. coli* W Δ*cscR* were inoculated as in Figure 2 and grown for 24 hours, then plated on solid ^COB^BG-11 agar. Plates were incubated in constant light until visible growth was apparent, whereupon green colonies were picked into individual wells containing liquid media and returned to the incubator. After (3-5) days of growth, cultures were visibly scored for the presence of cyanobacteria (green coloration) and plated on rich media to determine the presence or absence of heterotrophs (e.g. lower left panel: “X” mark wells containing cyanobacteria where heterotrophs were lost). Percentages of the wells containing one or both prokaryote(s) are shown from 387 wells and 442 wells in panel B.

**
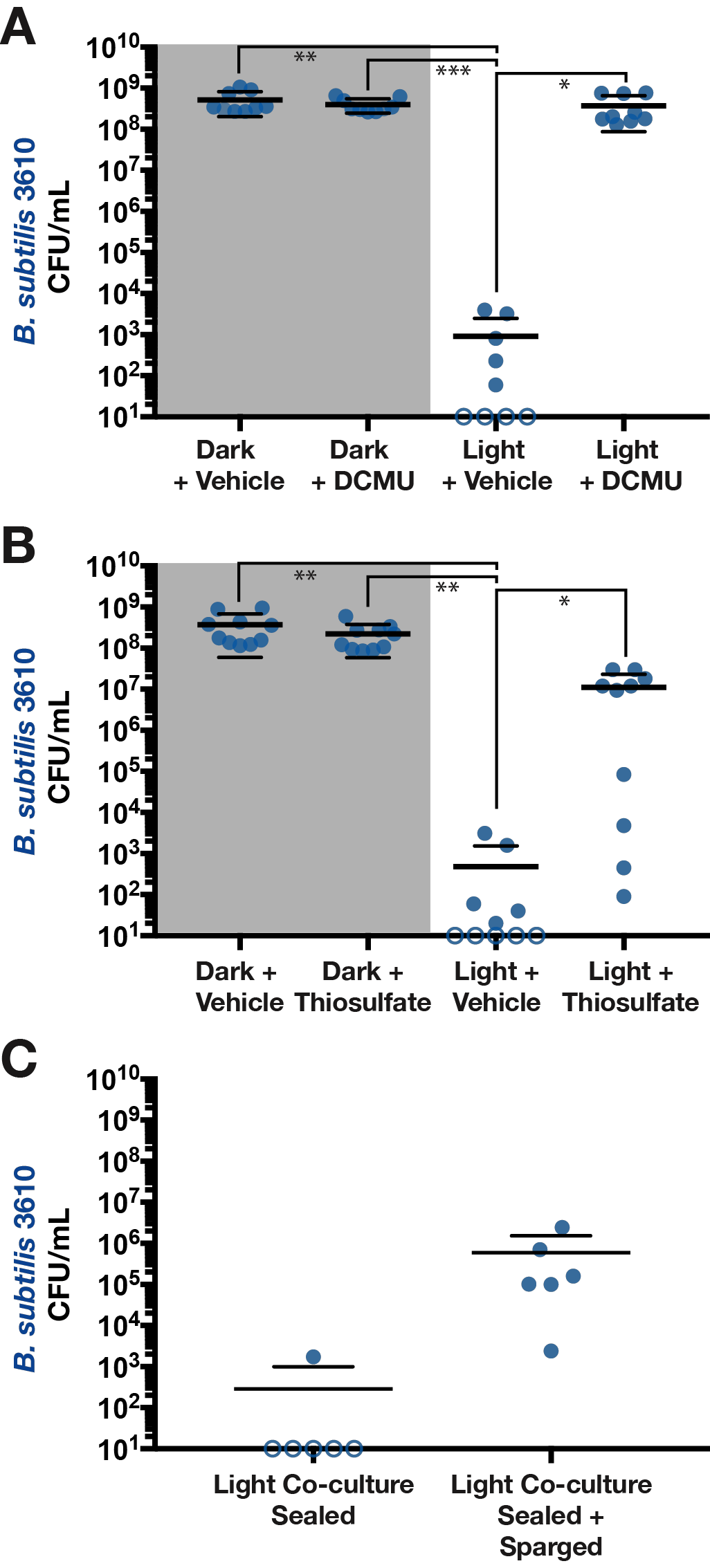
**

**Fig. S10 Hyper-oxygenation of shared media contributes to the inhibitory effect of *S. elongatus* on *B. subtilis* in co-culture.**

Co-cultures of *B. subtilis* 3610 and high density *S. elongatus* (6.6x10^8^cells/mL) were inoculated and grown with supplemented sucrose (2%) in light or dark (grey). A) The oxygen evolution Photosystem II inhibitor DCMU, or vehicle control was added to initial cultures. B) The antioxidant thiosulfate or vehicle control was added to initial cultures. After 12 hours, *B. subtilis* 3610 CFU/mL were determined. C) Co-cultures were prepared as above and parent cultures were split into equal volumes into two sealed containers. One container was sealed with atmospheric oxygen levels. The second was sparged with gas (12:10:82 H_2_:CO_2_:N_2_) to deplete oxygen. Viable *B. subtilis* CFUs were determined after 12 hours in the light. Biological replicates are represented by points on the graph; hollow circles on the x-axis represent replicates in which no colonies grew. Thick horizontal lines represent the average measurement for each condition while thin horizontal lines represent one standard deviation from the mean. Repeat measure one-way ANOVA was used to determine significant deviation between means. The Holm-Sidak multiple comparison test was used to compare all conditions to the light + vehicle control. P-values are denoted with asterisks: * 0.01 to 0.05, ** 0.001 to 0.01, *** 0.0001 to 0.001.

**
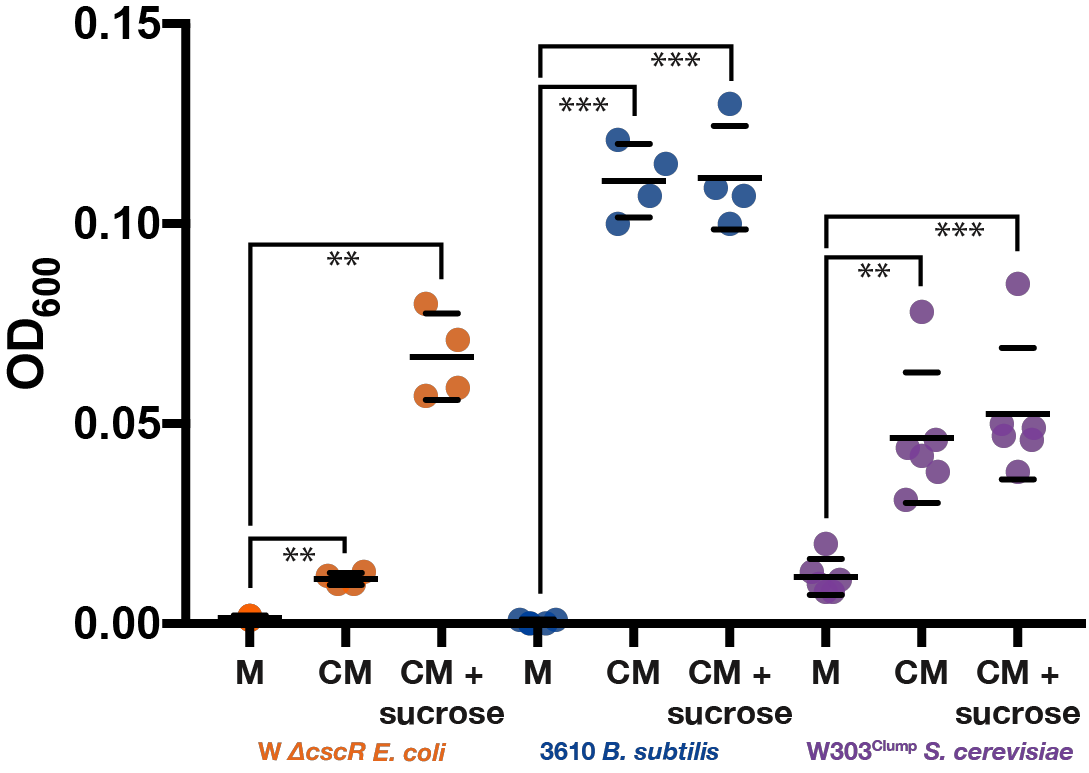
**

**Fig. S11 Heterotroph growth in media conditioned by *S. elongatus.***

Indicated heterotrophic strains were grown in fresh or cyanobacteria-conditioned media in 96-well plates similar to Fig. 2C. To condition media (^CoB^BG-11 for prokaryotes; ^CoY^BG-11 for *S. cerevisiae*) was seeded with *cscB^+^ S. elongatus* at OD_750_ 0.5 and allowed to grow in constant light for 48 hours with 1mM IPTG to induce sucrose export. This media was then filtered to acquire conditioned media (CM). Sucrose (2%) was added to a portion of CM yielding CM + sucrose. Fresh media with no supplemented sucrose (M; ^CoB^BG-11 for prokaryotes; ^CoY^BG-11 for *S. cerevisiae*) was used as a negative control. Indicated heterotrophic species were inoculated into the respective media and microbial growth was measured after 24 hours of growth for bacteria and 48 hours of growth for *S. cerevisiae* of growth by measuring OD_600_. Biological replicates are represented by points on the graph. Long horizontal lines represent the average measurement for each condition while short horizontal lines represent one standard deviation from the mean. Repeat measure one-way ANOVA was used to determine significant deviation between means. Dunnett’s test was used to compare growth in conditioned medias with fresh media. P-values are denoted with asterisks: * 0.01 to 0.05, ** 0.001 to 0.01, *** 0.0001 to 0.001.


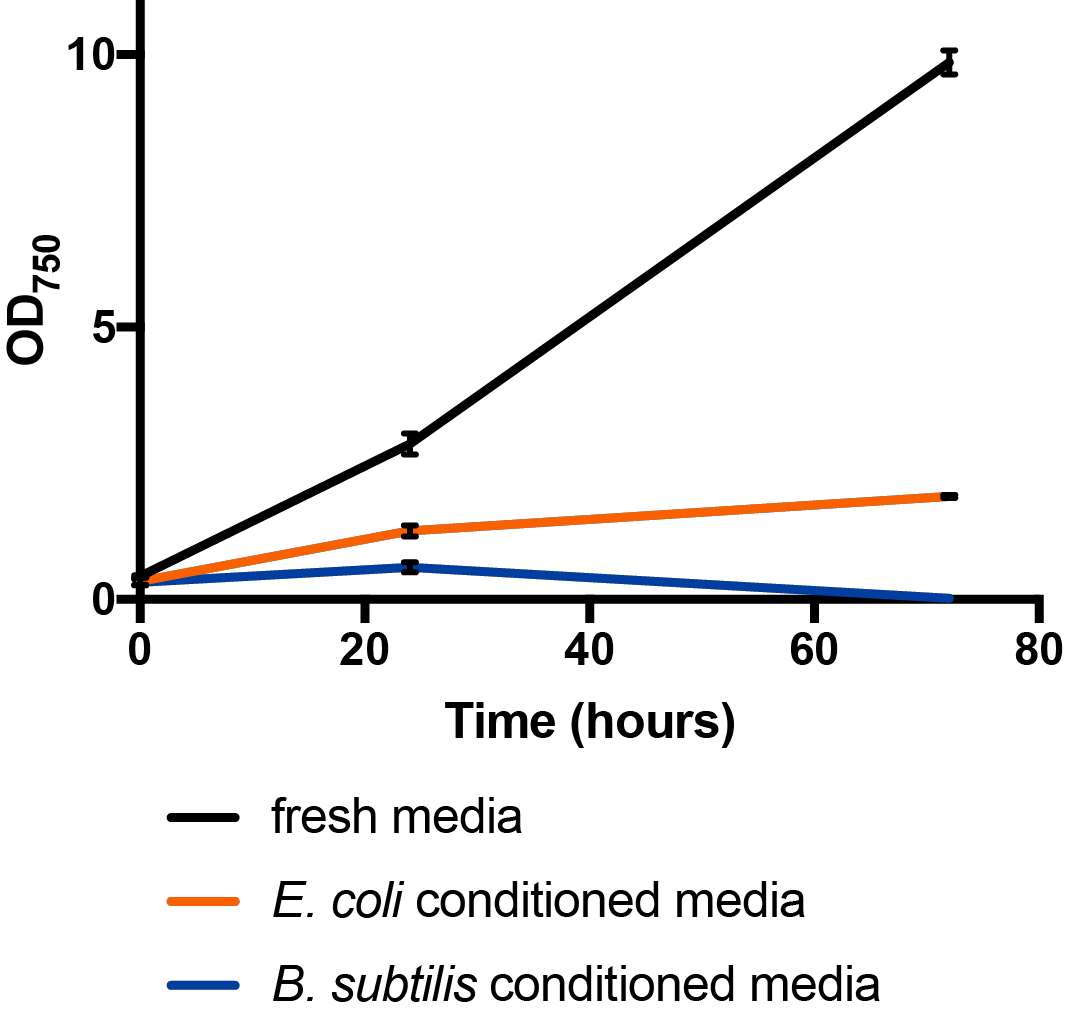


**Fig. S12 *S. elongatus* growth in media conditioned by heterotrophs.**

*E. coli* W Δ*cscR* and *B. subtilis* 3610 were inoculated in ^CoB^BG-11supplemented with 0.2% sucrose, then incubated at 35°C for 48 hours. At this point culture supernatants were filtered to remove heterotrophs. WT *S. elongatus* was inoculated in this heterotroph-conditioned media and grown under constant illumination*.* These cultures were tracked via OD_750_.

**Supplemental Material: Mathematical Framework**

**A. Mathematical framework describing interactions**

We summarized the interactions that were deliberately programmed in our synthetic co-cultures into a simple mathematical framework, illustrated in Fig. S13 and described in the subsequent sections: B. Initial Framework and C. Co-culture Framework. Briefly, the population of phototrophs, *P(t),* at time *t* can be approximated with a linear growth rate of $\mu_{p}$, based on the experimental data in Fig. 1B and 1C. The sucrose concentration, *S(t)*, is dependent both upon the production from cyanobacteria, *α*, and the consumption of sucrose by the heterotrophs, *β*. The heterotroph population, *H(t)*, is dependent on the maximal growth rate of each heterotroph on sucrose (*μ*_max_), the sucrose concentration at which the growth rate is half maximal (*S_halfmax_*), and the sucrose concentration (*S(t)*)[1, 2].


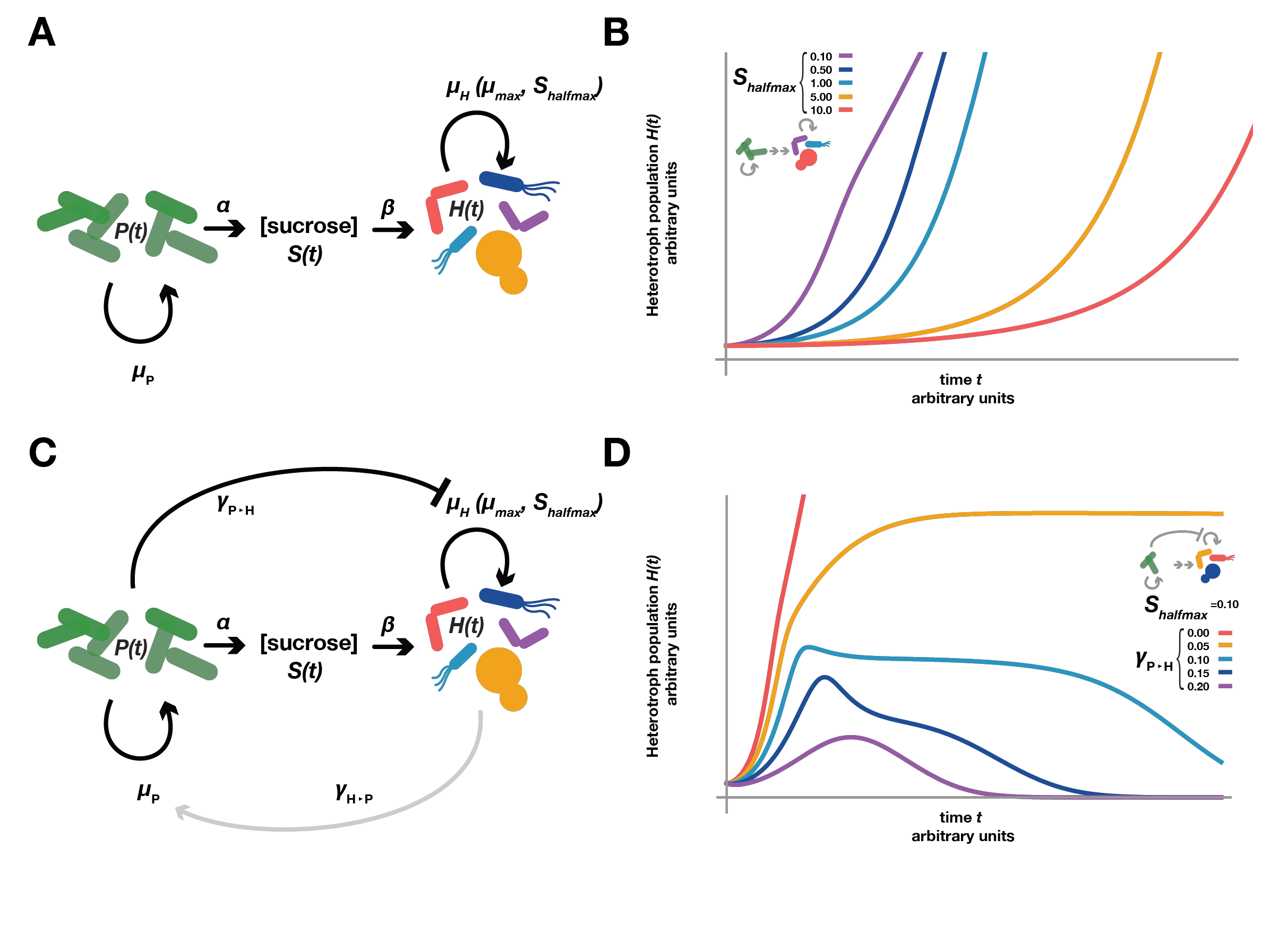


**Fig. S13 Mathematical Framework Describing Co-culture Interactions.**

(A, C) A mathematical framework summarizing consortia interactions we observe in this work is made up of: phototroph population (*P(t)*), phototroph growth rate ($\mu_{p}$), sucrose production rate by the phototroph ($\alpha$), sucrose necessary for a heterotroph cell to double ($\beta)$, the heterotroph population (*H(t)*), the heterotroph growth rate ($\mu_{H}$) which depends on the maximal growth rate when sucrose is not limiting ($\mu_{max})$and the sucrose concentration at which the growth rate is half maximal ($S_{halfmax}$), and the interaction terms describing the effects phototrophs haves on heterotrophs ($\gamma_{P⊣H})$, and heterotrophs have phototrophs ($\gamma_{H\to P}$). (A, B) When interaction terms $(\gamma_{P⊣H} \& \gamma_{H\to P})$ are set to zero, and all other variables are held constant, decreases in $S_{halfmax}$ increase growth and decrease time before the onset of heterotrophic growth. (C, D) Adding and tuning the $\gamma_{P⊣H}$ interaction term stops the monotonically increasing heterotroph growth when other variables are held constant; at high enough values, the heterotrophic population can decrease and/or growth is entirely eliminated. Changes in $\gamma_{H\to P}$(grey arrows in C, set to a value of ‘0’ in panel D) can also lead to feedback that increases the complexity of heterotrophic growth when other variables are held constant. As $\gamma_{H\to P}$increases there is a more pronounced drop in heterotroph concentration after the initial rise.

We added variables to this framework to represent the additional observed interactions between phototrophs and heterotrophs (Fig. 6C): cyanobacterial inhibition of heterotrophic viability (Fig. 3A) and heterotrophic stimulation of cyanobacterial growth (Fig. 3E). The inhibitory effect of cyanobacteria on heterotroph, *γ_P⊣H_*, scales as a function of the phototroph density *P(t)*, and negatively influences the heterotroph population *H(t)*. The growth-promoting effect of heterotrophs on cyanobacterial growth, *γ_H→P_*, is represented as a constant that influences the cyanobacterial growth rate, *μ_p_*, as we have not shown this effect to be density-dependent.

Although limited to being a conceptual tool, this simplistic mathematical framework can recapitulate aspects of the complex interactions we observe in our experiments (Fig. S13). For example, when the interaction terms, *γ_P⊣H_* and *γ_H→P_*, are set to 0 and all other parameters are held constant (details in section C. Co-culture Framework), increasing sucrose utilization (i.e. decreasing *S*_halfmax_) results in an earlier rise in heterotrophic population (Fig. S13B). This is consistent with data from *S. cerevisiae* co-cultures, as the W303 strain exhibits a high S_halfmax_ (Fig. 2C) and does not exhibit growth within co-culture (Fig. 2B) while the W303^Clump^ strain has a lower S_halfmax_ (Fig. 2C), and demonstrates growth within co-cultures (Fig. 2D).

More interestingly, the addition of the interaction term *γ_P⊣H_* changes the shape of the heterotroph population function when all other parameters are constant; at higher values the model predicts an initial rise in heterotrophic density followed by a subsequent decline (Fig. S13D). This is consistent with instances in which the heterotroph species are shown to be fairly sensitive to the inhibitory effects of cyanobacterial metabolism in the light (demonstrated in Fig. 3A-D), and recapitulates experimental co-culture dynamics we observe with *S. cerevisiae* and *B. subtilis* (Fig. 2A&D).

**Initial Framework**

Axenic cyanobacterial growth is approximated as linear, as observed in 48 hour batch co-cultures (Fig. 1B), by Equation 1:

|  | $P\left( t \right)=\mu_{p}t+P(0)$ | (1) |
| --- | --- | --- |

where *P(t)* is the phototroph population at time *t*, $\mu_{p}$ is the growth rate of the phototroph, and *P(0)* is the initial inoculum of phototrophs. All variables and functions are summarized in Table S1. The growth rate, $\mu_{p}$, is experimentally determined and differs in the presence or absence of IPTG, which induces *cscB* expression and diverts cellular resources from growth to sucrose secretion (Fig. 1B,1C, & S1). Experimentally determined values are summarized in Table S2.

**Table S1 Function and variable key.**

| **Variables & Functions** | **Definition** |
| --- | --- |
| *t* | Time |
| *P(t)* | Phototroph population at time t |
| $\mu_{p}$ | Phototroph growth rate |
| *S(t)* | Sucrose concentration at time t |
| $\alpha$ | Sucrose production rate by the phototroph |
| *H(t)* | Heterotroph population at time t |
| $\mu_{H}$ | Heterotroph growth rate |
| $\beta$ | Coefficient of proportionality |
| $\mu_{max}$ | Maximal heterotroph growth rate in sucrose |
| $S_{halfmax}$ | Sucrose concentration at which heterotroph growth rate is half maximal |
| $\gamma_{P⊣H}$ | Interaction coefficient describing phototroph interaction with heterotroph growth |
| $\gamma_{H\to P}$ | Interaction coefficient describing heterotroph interaction with phototroph growth |

**Table S2 Experimentally Determined Values for Initial Conditions in S14 and S15.**

| **Variables & Functions** | **Values** |
| --- | --- |
| *t* | 0 to 48 hours |
| *P(0)* | 1.000 x 10^8^ cells per mL |
| $\mu_{p}$ | Without IPTG: 1.785 x 10^7^ cells per hour  With IPTG: 1.021 x 10^7^ cells per hour |
| *S(0)* | 0 mg per mL |
| $\alpha$ | Without IPTG: 6.4412 x 10^-12^ mg per phototroph per hour  With IPTG: 3.9253 x 10^-11^ mg per phototroph per hour |

Sucrose production from cyanobacteria, as measured in Fig. 1C, can be approximated by Equation 2:

|  | $S'\left( t \right)=\alpha P(t)$ | (2) |
| --- | --- | --- |

where the sucrose concentration, *S(t)*, changes at a rate determined by the sucrose production rate, $\alpha$, and the phototroph population, *P(t)*, at time t. Equations 1 and 2, with variables set to experimentally determined values, are sufficient to simulate Fig. 1B and Fig. 1C, shown in Fig. S14 and S15, respectively.


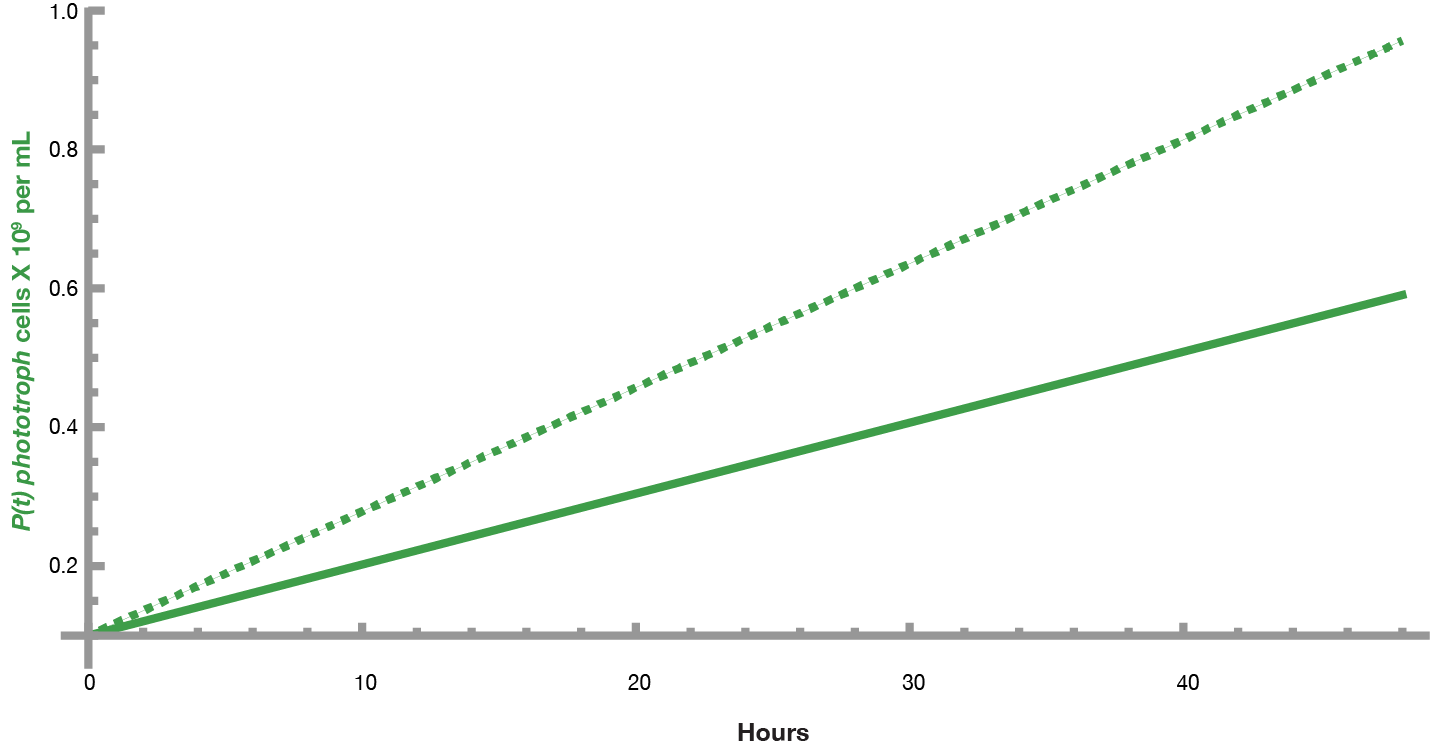


**Fig. S14 Model approximation of axenic phototroph growth.**

Using equation 1, axenic phototroph growth is approximated. The initial conditions and parameters used to generate this graph: *P(0)* is 1x10^8^ cells/mL, $\mu_{p}$ without IPTG (dashed line) was 1.785 x10^7^ hours^-1^,$\mu_{p}$ with IPTG (solid line) was 1.022 x10^7^ hours^-1^.


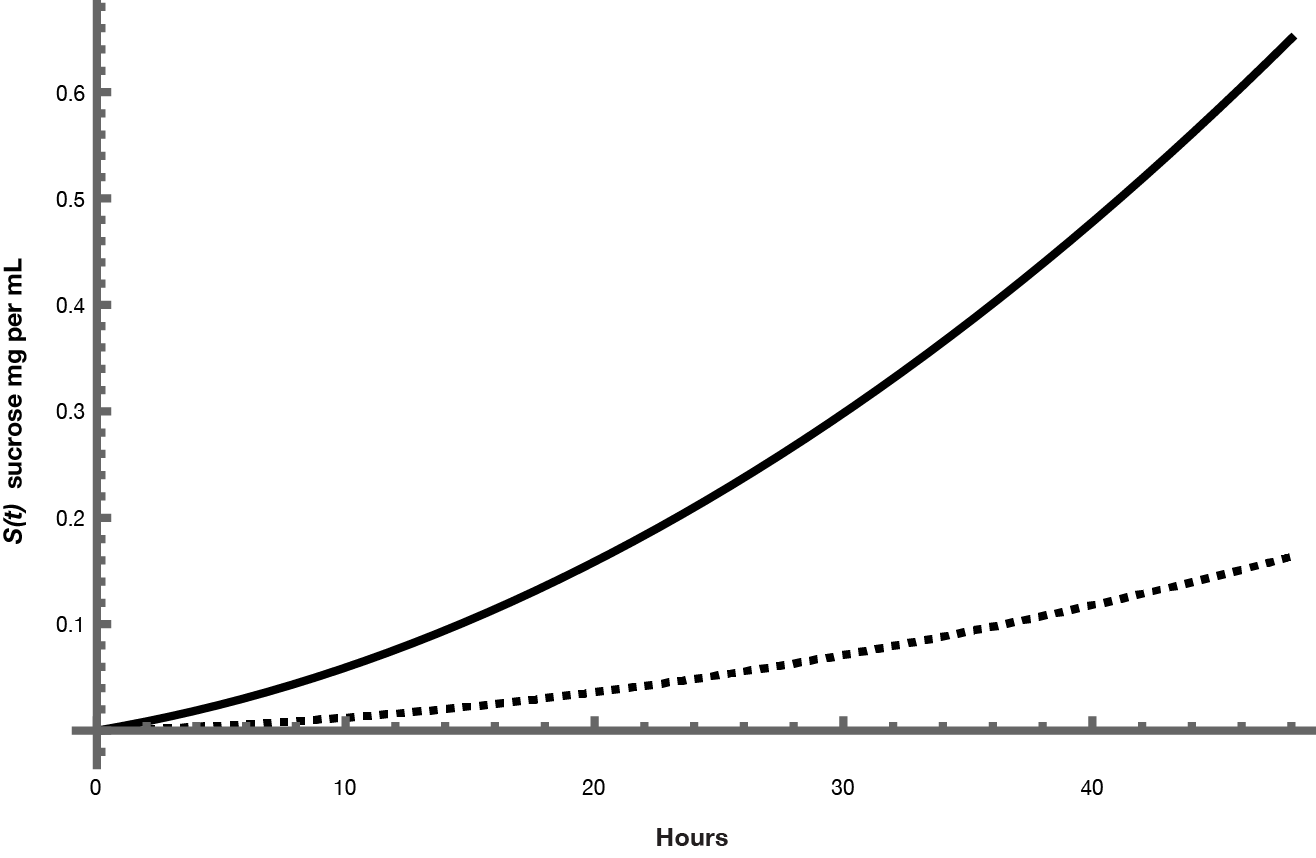


**Fig. S15 Model approximation of secreted sucrose from axenic phototroph.**

Using equations 1 & 2, sucrose production from the phototroph with (solid line) and without (dashed line) is approximated. The initial conditions and parameters used to generate this graph: *P(0)* is 1x10^8^ cells/mL, $\mu_{p}$ without IPTG is 1.785 x10^7^ hours^-1^,$\mu_{p}$ with IPTG is 1.022 x10^7^ hours^-1^, *S(0)* is 0 mg/mL, $\alpha$ without IPTG is 6.4412 x 10^-12^ mg per phototroph per hour, $\alpha$ with IPTG is 3.9253 x 10^-11^ mg per phototroph per hour.

To approximate heterotroph growth, the Monod Equation was used yielding:

|  | $H'\left( t \right)=\mu_{H}H(t)$ | (3) |
| --- | --- | --- |
|  | $\mu_{H}=\mu_{max} \frac{S(t)}{S_{halfmax}+S(t)}$ | (4) |

where the heterotroph population, *H(t)*, is dependent on the growth rate,$\mu_{H}$. That growth rate, $\mu_{H}$, is determined from the maximal growth rate of the heterotroph when sucrose is not limited ($\mu_{max}$), the concentration of sucrose at which the growth rate is half maximal ($S_{halfmax}$), and the sucrose concentration are the time (*S(t)*).

Meanwhile, the sucrose concentration decreases proportionally with the growth rate. The coefficient of proportionally ($\beta$) in the amount of sucrose required for the creation of a new cell. This results in:

|  | $S^{'}(t)=-\beta H(t)\mu_{max} \frac{S(t)}{S_{halfmax}+S(t)}$ | (5) |
| --- | --- | --- |

**Co-culture Framework**

To make a framework for co-cultures, the sucrose concentration is a combination of equations 2 and 5.

|  | $S^{'}(t)=\alpha P(t)-\beta H(t)\mu_{max} \frac{S(t)}{S_{halfmax}+S(t)}$ | (6) |
| --- | --- | --- |

The density dependent impact of cyanobacteria impact on heterotroph can be implemented by altering equation 4 to:

|  | $\mu_{H}=\mu_{max} \frac{S(t)}{S_{halfmax}+S(t)}-\gamma_{P⊣H}P(t)$ | (7) |
| --- | --- | --- |

In which $\gamma_{P⊣H}$ is the interaction term between phototrophs and heterotrophs. This interaction was shown to be dependent on the phototroph population density, *P(t).*

In contrast, because we have not seen density dependence in respect to the positive effect of heterotrophs on phototroph growth, denoted by $\gamma_{H\to P}$, we chose to alter the cyanobacterial growth equation with the addition of this interaction coefficient to the growth rate:

|  | $P\left( t \right)={(\mu}_{p}+\gamma_{H\to P})t+P(0)$ | (8) |
| --- | --- | --- |

This means that the complete framework for co-culture can be summarized in equation 8, 3, 7, and 6.

|  | $P\left( t \right)={(\mu}_{p}-\gamma_{H\to P})t+P(0)$ | (8) |
| --- | --- | --- |
|  | $H'\left( t \right)=\mu_{H}H(t)$ | (3) |
|  | $\mu_{H}=\mu_{max} \frac{S(t)}{S_{halfmax}+S(t)}-\gamma_{P⊣H}P(t)$ | (7) |
|  | $S^{'}(t)=\alpha P(t)-\beta H(t)\mu_{max} \frac{S(t)}{S_{halfmax}+S(t)}$ | (6) |

**Exploration of parameter space in Fig. S11**

To explore the effects of varying parameters, the interaction coefficients were set to zero and the remaining variables were set to the values in Table S2. Arbitrary units were used due to the inability to determine experimental values for many of the variables.

**Supplemental References**

1. Monod J: *Recherches Sur La Croissance Des Cultures Bactériennes,*. Paris: Hermann & cie; 1942.

2. Smith H.L. *"*Bacterial Growth." *https://math.la.asu.edu/~halsmith/bacteriagrow.pdf* Department of Mathematics and Statistics, Arizona State University
